# Study on the Suitability of Concrete Constitutive Models for Perforation Simulation

**DOI:** 10.3390/ma17225562

**Published:** 2024-11-14

**Authors:** Jianxing Li, Yize Liu, Peiyu Li, Haifu Wang, Pengwan Chen

**Affiliations:** State Key Laboratory of Explosion Science and Technology, Beijing Institute of Technology, Beijing 100081, China; 3120215133@bit.edu.cn (J.L.); 3120230192@bit.edu.cn (Y.L.); 3120225098@bit.edu.cn (P.L.); pwchen@bit.edu.cn (P.C.)

**Keywords:** concrete constitutive model, perforation, numerical simulation, damage, crack

## Abstract

The choice of constitutive model significantly affects the accuracy of concrete perforation simulation. This study analyzes four concrete constitutive models, HJC, RHT, KCC, and TCK, focusing on their strength models, damage evolution, and strain rate effects. Combining the damage pattern and erosion cracks, the effectiveness of the four constitutive models in simulating the penetration of reinforced concrete targets is evaluated using LS-DYNA 11.0. The results indicate that the RHT and TCK models accurately depict the concrete damage and failure modes under the same test conditions. In contrast, the KCC and HJC models demonstrate superior capability in predicting the residual velocity of the projectile. Additionally, this study highlights the significant impact of the erosion parameters on the simulation results. This study offers a valuable reference for the application and parameter set of constitutive models in simulating concrete target perforation.

## 1. Introduction

Concrete is a crucial component of both civil and military protective structures. With the notable increase in armed conflicts and terrorist activities in recent years, the nonlinear dynamic response of concrete under extreme loads, such as high-speed impacts and blasts, has become a concern for researchers. Along with the direct impact of a projectile with high residual velocity, scabbing failure can occur near the structural rear surface due to the intensive stress wave and shear effect of the projectile, resulting in a concrete splash that endangers occupants’ personal safety [1]. Therefore, for concrete protective structures, estimating the failure in perforation should receive more attention.

The perforation test is a method commonly used to examine the performance of a concrete panel under high-speed projectile impact. Hanchak et al. [2] performed a series of 25.3 mm diameter projectile perforation tests on 178 mm thick normal-strength (48 MPa) and high-strength (140 MPa) concrete targets, and they determined that the residual velocity is not strongly affected by the compressive strength at a high impact velocity. Unosson and Nilsson [3] conducted a 75 mm diameter projectile perforation test on a 153 MPa concrete panel with a striking velocity of 620 m/s, verifying the numerical model. Kristoffersen et al. [4] experimentally determined the ballistic limit for C35, C75, and C100 concrete targets through a 20 mm diameter projectile impact test on a 50 mm thick concrete panel. Wu et al. [5] conducted a set of 25.3 mm diameter projectile perforation tests on monolithic and segmented concrete panels (100–300 mm in thickness with a compressive strength of 41 MPa), and they provided the cratering dimensions, projectile residual velocities, and decelerations in perforation. In the aforementioned work, the projectile residual velocity and concrete target failure (front cratering and back scrabbing) were considered key parameters used to assess the perforation resistance of concrete.

With the rapid refinement of simulation technology, the numerical method has become an effective tool for analyzing the failure mechanisms of concrete under dynamic loads. Numerous studies have shown that the accuracy of numerical simulation primarily depends on the selection of dynamic constitutive models of concrete [6]. Over the past few decades, researchers worldwide have conducted extensive studies on the mechanical properties of concrete under diverse dynamic loading conditions. Several dynamic constitutive models have been developed and improved, with HJC [7], RHT [8], KCC [9], and TCK [10] being the most widely used and studied in the field of perforation.

Due to the significant differences and diverse application ranges among these models, researchers have conducted comparative analyses of the application of constitutive models [11]. Li [12] comprehensively compared five commonly used concrete dynamic constitutive models and analyzed the characteristics and influences when selecting constitutive models from a theoretical perspective, but the research lacked analysis and verification of the numerical simulation. Cui et al. [13] discussed the basic mechanism of the dynamic mechanical properties of concrete and four common constitutive theoretical models in detail and found that the KCC model fully considered the factors affecting the constitutive properties of concrete at high strain rates, but the result of the damage simulation was not validated with tests. Abedini et al. [14] conducted a review of various concrete and steel models in LS-DYNA, evaluating the effects of simulating the impact response of reinforced concrete structures in different situations. However, the discussions concerning different working conditions lacked a consistent standard and further theoretical analysis. Zhang et al. [15] compared the HJC with RHT models using penetration simulation tests and modified the failure strength parameters of the concrete constitutive model. However, the study was limited to a comparative analysis of the projectile’s penetrating velocity, and there was no further discussion of the concrete failure mechanism. Du [16] performed a comparative study of three constitutive models of concrete under explosion impact and analyzed the different responses of the HJC, RHT, and KCC models under near-field explosion in detail. However, the study lacked a discussion of the damage caused by perforation.

In summary, the existing comparisons of concrete constitutive models for perforation simulation are limited to HJC, RHT, and KCC models. A comparative study of the TCK model in relation to other models has not been reported. In light of this, to clarify the suitability of concrete constitutive models for simulating penetration problems, this study theoretically analyzes and reviews the characteristics of four commonly used concrete dynamic constitutive models: HJC, RHT, KCC, and TCK. Combined with LS-DYNA, the numerical simulation of reinforced concrete penetration experiments is conducted to analyze and compare the dynamic response and failure mechanism of the four constitutive models when describing penetration problems. The research results are of great significance for selecting constitutive models of concrete for use in penetration simulation.

## 2. Basic Analysis of Commonly Used Concrete Constitutive Models

In this section, a basic analysis of the four constitutive models is presented. Firstly, the theory of the four models is presented in Section 2.1. In Section 2.2, a comparative analysis of the models is performed in terms of the strength surface theory, damage evolution, and strain rate effect. Finally, a concise overview of the distinctive properties of each model is provided.

### 2.1. Theoretical Model

#### 2.1.1. HJC Model

Holmquistet al. [7] proposed the Holmquist–Johnson–Cook (HJC) model by referring to the basic equations of the Johnson–Cook model. In the case of compression, the yield surface of the HJC model is defined as follows:

(1) $$\sigma_{eq}^{*}=\left[A(1-D)+Bp^{*N}\right]\left(1+C\ln{\dot{\varepsilon }}^{*}\right)\leq S_{\max},p^{*}\geq 0$$

where $\sigma_{eq}^{*}$ is the normalized equivalent strength, $\sigma_{eq}^{*}=\sqrt{3J_{2}}/f_{c}$, *J*_2_ is the second invariant of the deviational stress tensor, and *f*_c_ is the unconfined uniaxial compressive strength. *S*_max_ is the normalized ultimate compressive strength, $p^{*}$ is the normalized pressure, $p^{*}=p/f_{c}$, *p* is the real pressure, ${\dot{\varepsilon }}^{*}$ is the dimensionless strain rate, ${\dot{\varepsilon }}^{*}=\dot{\varepsilon }/{\dot{\varepsilon }}_{0}$, and $\dot{\varepsilon }$ is the true strain rate, ${\dot{\varepsilon }}_{0}=1s^{-1}$. The material constants *A*, *B*, *N*, and *C* represent the normalized cohesion strength, the normalized pressure hardening coefficient, the pressure-hardening index, and the strain rate coefficient, respectively.

Damage factor *D* is defined as

(2) $$D=\sum \frac{\Delta \varepsilon_{p}+\Delta \mu_{p}}{\varepsilon_{p}^{f}+\mu_{p}^{f}}$$

where $\Delta \varepsilon_{p}$ and $\Delta \mu_{p}$ are the effective plastic strain increment and plastic volume strain increment in one time step, respectively. $\varepsilon_{p}^{f}+\mu_{p}^{f}$ is the total plastic strain, which is determined using the following formula:

(3) $$\varepsilon_{p}^{f}+\mu_{p}^{f}=D_{1}{\left(p^{*}+T^{*}\right)}^{D_{2}}\geq \;\mathrm{EFMIN}\;$$

where $T^{*}=T/f_{c}$, where $T^{*}$ is the normalized tensile strength, and *T* is the unconfined uniaxial tensile strength. EFMIN is a material constant for inhibiting the fracture caused by a weak tensile wave. *D*_1_ and *D*_2_ represent damage constants.

For tension, the yield surface equation is

(4) $$\sigma_{eq}^{*}=Ap^{*}/\left[T^{*}(1-D)\right]+A,p^{*}<0$$

**Figure 1:**
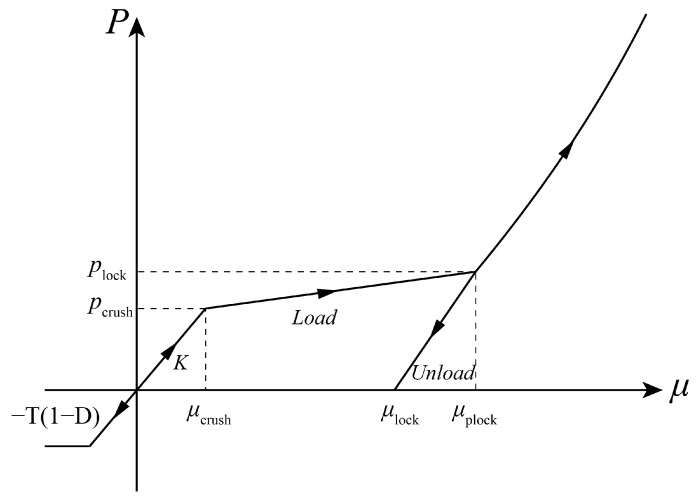
The EOS of the HJC model.

The equation of state (EOS) that represents the relationship between volume stain *μ* and pressure *p* is illustrated in Figure 1. Compression is divided into three stages. The first stage is the linear elastic phase. The second stage is the transition phase, which means when the initial compaction pressure *p_crush_* is reached, the voids inside the concrete material are gradually crushed to produce plastic deformation. The third stage is the full compaction phase, when the pressure exceeds *p_lock_*, air voids completely vanish, and the relationship in this stage is expressed by polynomials. The EOS in tension is elastic–perfectly plastic with damage factor *D*.

#### 2.1.2. RHT Model

Riedel et al. [8] improved the HJC model and proposed the Riedel–Hiermaier–Thoma (RHT) model by introducing three strength surfaces, namely the failure surface, elastic yield surface, and residual strength surface.

The failure surface is defined as

(5) $$Y_{\mathrm{fail}\;}(p,\theta ,\dot{\varepsilon })=f_{c}Y_{\mathrm{TXC}\;}^{*}\left(p/F_{\mathrm{rate}\;}\right)R_{3}(\theta )F_{\mathrm{rate}\;}(\dot{\varepsilon })$$

where *f_c_* is the uniaxial compressive strength, $Y_{\mathrm{TXC}\;}^{*}$ is the compressive meridian, *p* is the current pressure, *F*_rate_ is the strain rate correlation function, and *R*_3_(*θ*) is the Lode Angle function.

The elastic yield surface is defined as

(6) $$Y_{\mathrm{elastic}\;}(p,\theta ,\dot{\varepsilon })=f_{c}Y_{\mathrm{TXC}\;}^{*}\left(p/F_{\mathrm{rate}\;}/F_{\mathrm{elastic}\;}\right)R_{3}(\theta )F_{\mathrm{rate}\;}(\dot{\varepsilon })F_{\mathrm{elastic}\;}F_{\mathrm{cap}\;}$$

where *F*_elastic_ is the ratio of the yield strength to failure strength, and *F*_cap_ is the cap function of the yield surface, which is used to describe the plastic volume deformation of geotechnical materials caused by hydrostatic pressure.

The residual surface is defined as

(7) $$Y_{\mathrm{residual}\;}(p)=f_{c}B{\left(p^{*}\right)}^{M}$$

*B* and *M* are parameters of the residual surface, which can be obtained through experiments.

The yield surface in the hardening state before failure is determined using the interpolation between the failure surface and the elastic yield surface.

(8) $$Y_{\mathrm{hard}\;}=Y_{\mathrm{elastic}\;}+\frac{\varepsilon_{p}}{\varepsilon_{p}^{\mathrm{hard}\;}}\left(Y_{\mathrm{failure}\;}-Y_{\mathrm{elastic}\;}\right)$$

The yield surface in the softening state is determined using the interpolation between the failure surface and the residual strength surface to reflect the process of damage.

(9) $$Y_{\mathrm{frac}\;}=(1-D)Y_{\mathrm{failure}\;}+DY_{\mathrm{residual}\;}$$

When the strengthened yield surface reaches the ultimate strength, the damage factor *D* is calculated.

(10) $$D=\sum \frac{\Delta \varepsilon_{p}}{\varepsilon_{p}^{\mathrm{failure}\;}}\;$$

(11) $$\varepsilon_{p}^{\mathrm{failure}\;}=D_{1}{\left(p^{*}+T^{*}\right)}^{D_{2}}\geq \varepsilon_{f,\;\min\;}$$

where $\Delta \varepsilon_{p}$ is the equivalent plastic strain increment, $\varepsilon_{p}^{\mathrm{failure}\;}$ is the failure plastic strain, $D_{1}$ and $D_{2}$ are the damage parameters, $p^{*}+T^{*}$ is the normalized current hydrostatic pressure, and $\varepsilon_{f,\;\min\;}$ is the minimum failure strain.

The strain rate effect of the RHT model is described as follows:

(12) $$DIF(\dot{\varepsilon })=\left\{\begin{array}{l} {\left(\frac{\dot{\varepsilon }}{{\dot{\varepsilon }}_{0c}}\right)}^{\beta_{c}}p\geq 0\\ {\left(\frac{\dot{\varepsilon }}{{\dot{\varepsilon }}_{0t}}\right)}^{\beta_{t}}p\leq 0 \end{array}\right.$$

The EOS of the RHT model also divides the compression stage into three zones. Differently from the HJC model, the plastic transition phase (the second stage) adopts the *p*-*α* EOS to reflect the volume change characteristics of pore compaction in concrete. The third stage adopts the polynomial EOS to describe the fully compacted material.

#### 2.1.3. KCC Model

The KCC model was developed using the LLNL model in DYNA3D [9]. The model takes three independent strength surfaces, namely the initial yield strength surface $\Delta \sigma_{y}$, maximum strength surface $\Delta \sigma_{m}$, and residual strength surface $\Delta \sigma_{r}$. The basic equation is as follows:

(13) $$\Delta \sigma_{y}=\left\{\begin{array}{l} a_{0y}+p/\left(a_{1y}+a_{2y}p\right),p\geq f_{yc}/3\\ 1.35T+3p\left(1-1.35T/f_{yc}\right),0\leq p\leq f_{yc}/3\\ 1.35(p+T),p\leq 0 \end{array}\right.$$

(14) $$\Delta \sigma_{m}=\left\{\begin{array}{l} a_{0}+p/\left(a_{1}+a_{2}p\right),p\geq f_{c}/3\\ (1.5/\Psi )(p+T),\left\{0\leq p\leq f_{c}/3\right\}\;\mathrm{or}\;\left\{\lambda \leq \lambda_{m}\;\mathrm{and}\;-T\leq p\leq f_{c}/3\right\}\\ 3(p/\eta +T),p\leq 0\;\mathrm{and}\;\lambda >\lambda_{m} \end{array}\right.$$

(15) $$\Delta \sigma_{r}=a_{0f}+p/\left(a_{1f}+a_{2f}p\right)$$

where *a*_0*y*_, *a*_1*y*_, *a*_2*y*_, *a*_0*f*_, *a*_1*f*_, and *a*_2*f*_ are material constants determined using triaxial compression test data. *f_yc_* is the initial yield strength, generally 0.45 *f_c_*, *f_c_* and *T* are the unconfined uniaxial compressive strength and tensile strength, *p* is the hydrostatic pressure, and *Ψ* denotes the tensile-to-compressive meridian ratio.

The failure surface is divided into two stages, strain hardening and strain softening.

(16) $$\Delta \sigma =\sqrt{3J_{2}}=\left\{\begin{array}{l} r^{\prime }\left[\eta \left(\Delta \sigma_{m}-\Delta \sigma_{y}\right)+\Delta \sigma_{y}\right],\;\mathrm{strain}\;\mathrm{hardening}\;\\ r^{\prime }\left[\eta \left(\Delta \sigma_{m}-\Delta \sigma_{r}\right)+\Delta \sigma_{r}\right],\;\mathrm{strain}\;\mathrm{softening}\; \end{array}\right.$$

where *J*_2_ is the second deviatoric stress invariant, *r*′ is the ratio of the current meridian to the compression meridian, and *η* is related to the degree of damage *λ*. *η* increases from 0 to 1 in the strain hardening stage and decreases from 1 to 0 in the strain softening stage after the damage begins. The KCC model calculates the compression and tensile damage separately, and the *λ* is determined by

(17) $$\lambda =\sum \left\{\begin{array}{l} \Delta {\bar{\varepsilon }}_{p}/{\left(1+p/T\right)}^{b1},p>0\\ \Delta {\bar{\varepsilon }}_{p}/{\left(1+p/T\right)}^{b2},p\leq 0 \end{array}\right.$$

where $\Delta {\bar{\varepsilon }}_{p}$ is the effective plastic strain increment in each time step, and *b*_1_ and *b*_2_ are compressive softening coefficient and tensile softening coefficient, respectively.

The strain rate effects of the strength model and damage evolution equation are defined by

(18) $$\Delta \sigma =r_{f}\Delta \sigma \left(p/r_{f}\right),\;\lambda =\lambda \left(p/r_{f}\right)/r_{f}$$

where *r_f_* is the dynamic increase factor (DIF).

The KCC model uses *EOS_TABULATED_COMPACTION in LS-DYNA to control the relationship between current pressure *p* and volume strain *μ*.

(19) $$p=C(\mu )+\gamma_{0}T(\mu )E_{0}$$

where *E*_0_ is the energy per unit volume, and *γ*_0_ is the specific heat capacity. *C*(*μ*) and *T*(*μ*) indicate pressure and temperature related to volume strain. The *p*-*μ* curve is similar to the HJC model.

#### 2.1.4. TCK Model

The TCK model adopted the theory of microcracks derived by Budiansky and O’Connell [17]. The stress–strain relationship of the model is as follows:

(20) $$P=3K(1-D)\varepsilon_{v}$$

(21) $$S_{ij}=2G(1-D)e_{ij}$$

where *P* is the volume stress, *K* is the initial volume modulus, *ε_v_* is the volume strain, *S_ij_* is the deviational stress, *G* is the initial shear modulus, *e_ij_* is the partial strain tensor, and *D* is the damage factor, which is determined by

(22) $$D=\frac{16}{9}\frac{1-v^{2}}{1-2v}C_{d}$$

where *v* is the Poisson ratio, and *C_d_* is the crack density parameter per unit volume.

(23) $$C_{d}=\frac{5}{2}k\varepsilon_{v}^{m}{\left(\frac{K_{1C}}{\rho C}\right)}^{2}{\dot{\varepsilon }}_{v\max}^{-2}$$

where *k* and *m* are material constants related to the tensile strain rate, *K*_IC_ is the fracture toughness, *ρ* is the material density, *C* is the material uniaxial elastic wave velocity, and ${\dot{\varepsilon }}_{v\max}$ is the maximum volume strain rate experienced by the element. Under compression, the TCK model assumes that the material is elastic–perfectly plastic.

### 2.2. Comparison of Constitutive Model Theory

#### 2.2.1. Strength Surface

**Figure 2:**
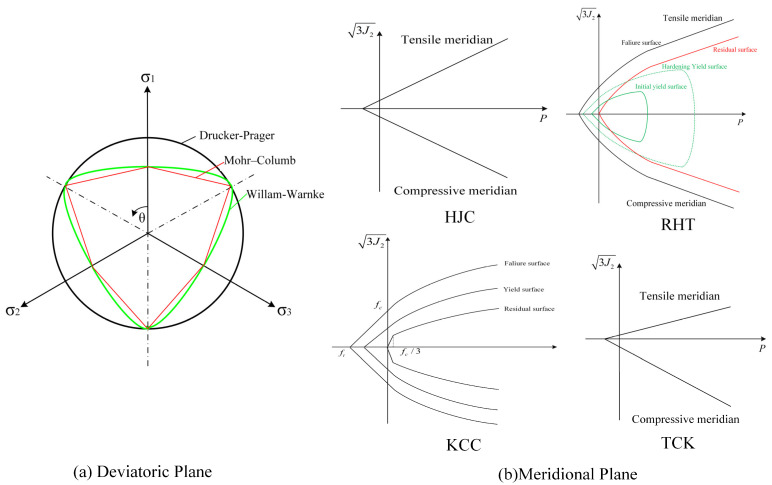
Strength surfaces in deviatoric and meridian planes.

The general strength criterion for concrete is defined as a function of the state of stress valid for concrete under general three-dimensional stress states, such as the Mohr–Coulomb criterion, Drucker–Prager criterion, Willam–Warnke criterion, Ottosen criterion, etc. [18]. The deviatoric plane and meridional plane of the strength surface of the four models are shown in Figure 2.

The HJC model has a yield surface that uses the Drucker–Prager model without considering the effect of the third deviatoric stress invariant *J*_3_. The shape transformation, in which the deviatoric plane in the stress space changes from a triangle under low pressure to a circle under high pressure, is neglected. Therefore, it cannot capture the asymmetric properties in the tension and compression of concrete [19].

The failure surface of the RHT model is the Willam–Warnke criterion that contains the third deviatoric stress invariant, which can truly reflect the properties of concrete [20]. However, as shown in Formula (7) in Section 2.1.2, the residual strength is an exponential function of the pressure without considering the effect of the Lode Angle and the third deviatoric stress invariant. When the loading path corresponds to a Lode Angle of zero degrees on the tensile meridian (biaxial compression state), it leads to the phenomenon wherein the residual strength is greater than the initial yield strength [21,22].

Different from the RHT model, the influences of *J*_3_ are considered in all three strength surfaces of the KCC model. The relationship of the tension–compression meridian ratio is expressed by piecewise function for a more accurate description [23].

The compression model of the original TCK is elastic–perfectly plastic, which leads to difficulty in reflecting the plastic mechanical behavior. Although Chen introduces the Drucker–Prager criterion into the TCK model in [24], the Drucker–Prager criterion is still symmetrical in its tensile–compressive meridian, which is not suitable for characterizing the mechanical properties of concrete. Therefore, the Mohr–Columb criterion is employed in the TCK model in this study.

#### 2.2.2. Damage Evolution Model

Defects in the material at the microstructure level are termed “damage”, such as micro-voids and microcracks. The nonlinear stress–strain behavior of concrete is mainly due to the propagation and nucleation of microcracks. To deal with the stiffness degradation and softening of concrete, Lamai [25] proposed the strain equivalence hypothesis, which is defined as

(24) $$\sigma =(1-D)\sigma_{i}$$

*σ* is the true stress of damaged material, and *σ_i_* is the stress of undamaged material. The damage factor *D* is defined as follows:

(25) $$D=1-\frac{\Phi }{\Phi_{0}}$$

where Φ and Φ_0_ are the performance parameters of damaged and undamaged materials, such as the strength of concrete, Young’s modulus, density, and the evolution of microcracks and micropores.

**Figure 3:**
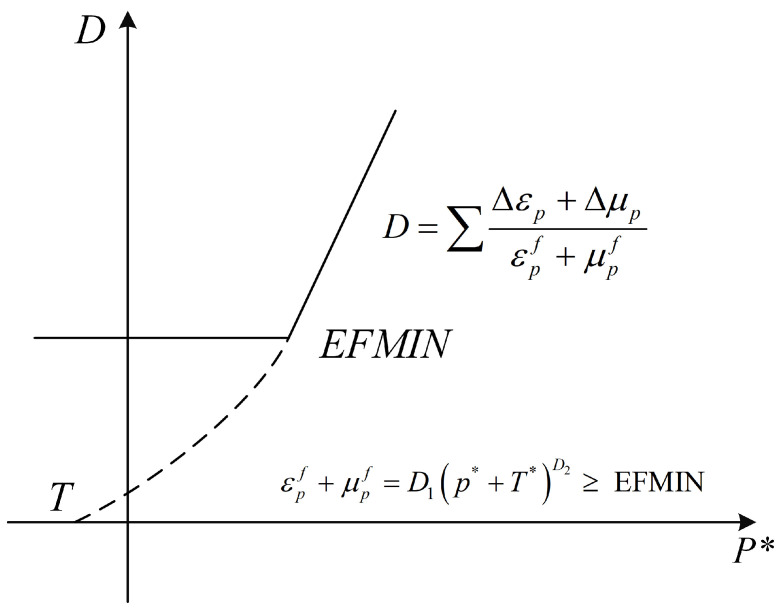
The damage evolution of HJC and RHT models.

The damage is accumulated by effective plastic strain in the HJC, RHT, and KCC models. The damage value of HJC is accumulated from both equivalent plastic strain and plastic volumetric strain; that is, the damage value starts accumulating when equivalent stress exceeds the failure surface. The damage evolution theory of the RHT model is the same as that of the HJC model, as shown in Figure 3.

**Figure 4:**
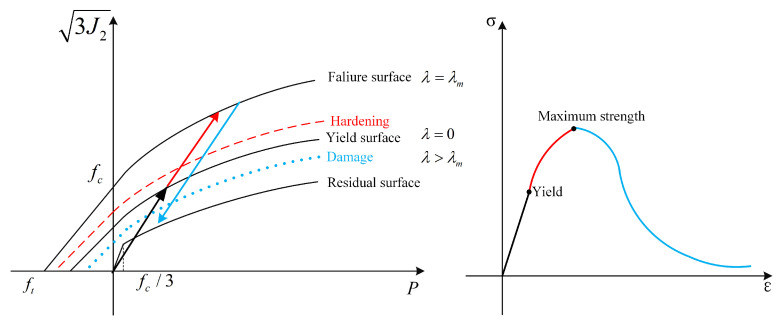
The damage evolution of the KCC model.

The plastic strain damage of the KCC model is controlled by *λ*. Statistics of *λ* begin when the material yields and the material enters the strain hardening stage. When the material reaches the ultimate strength (*λ* > *λ_m_*), the damage softening stage begins, as shown in Figure 4. Although the calculation of damage in the KCC model is relatively comprehensive, there is no direct expression provided in LS-DYNA, and damage is commonly described through the effective plastic strain. The damage evolution parameters of the KCC model are automatically generated in LS-DYNA based on the strength parameters.

The TCK model assumes that there are a large number of penny-shaped microcracks inside the material. According to the classical damage theory of the crack generation process, the failure progress is represented by the degradation of the bulk modulus *K*.

(26) $$\frac{\bar{K}}{K}=1-\frac{16}{9}\left(\frac{1-v^{2}}{1-2v}\right)C_{d}$$

where *K* is the original constant for the undamaged material, and the effective bulk modulus given by Formula (26) vanishes as the crack density approaches a critical value of 9/16. It seems plausible that, as the microcracks grow and intersect to the point that slightly more than one-half of the material volume is composed of cracks, the material stiffness is completely lost. After reaching the threshold value, the element is deleted to characterize the tensile failure of concrete. The compression damage and failure are controlled by a critical value of effective plastic strain.

#### 2.2.3. Strain Rate Effect

The mechanism of the strain rate effect of concrete can be attributed to the viscous effect, inertial effect, and crack evolution. The main role of the viscous effect and inertial effect is limiting the localization of microcracks and the propagation of macrocracks. When the strain rate is lower than 1/s, the viscous effect plays a leading role in the dynamic mechanical properties of concrete, which is related to the water content of concrete [26,27,28,29,30]. When the strain rate is greater than 10/s, the inertial effect dominates [31], and the lateral restraint caused by the Poisson effect limits the deformation of concrete. The crack evolution of concrete varies greatly at different strain rates. Under quasi-static load, microcracks grow and expand slowly along the weak surface of the combination of aggregate and mortar, while under high strain rate conditions, there might not be enough time for cracks to find the weakest surface, and new cracks are forced to pass directly through the high-strength aggregate [32,33,34], so the macroscopic strength of concrete is improved.

**Figure 5:**
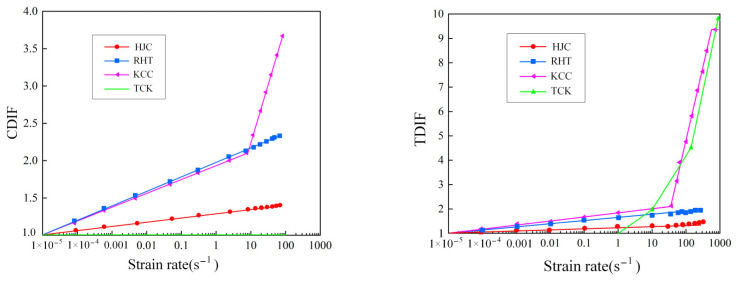
Compressive and tensile strain rate effects.

The strain rate effect is represented by the isotropic expanding method in the four models. Figure 5 shows the compression and tensile strain rates of the four models. The HJC model uses the natural logarithm form of the equivalent strain rate, the RHT model uses the exponential function form of the equivalent strain rate to describe the strain rate strengthening effect, and the strain rate enhancement factor of KCC can be determined through tests and freely input, generally using CEB code [34]. The TCK model has no compressive strain rate effect, and the tensile strain rate is derived by tensile fracture stress [35]. It should be noted that DIF relationships in models are all obtained by directly fitting test data, so the DIF includes the dynamic characteristics of materials and the strength increase caused by structural effects [36,37,38], which may lead to errors due to the test conditions and sample size.

To summarize, the RHT and KCC models comprehensively describe the mechanical properties of concrete. The HJC model is not accurate in describing the tensile properties of concrete, while the TCK model compression strength model is relatively simplified. In terms of damage theory, the basic principles of HJC, RHT, and KCC models are similar. The KCC model considers tensile and compressive damage separately, while only one damage factor is used, and the value cannot be directly depicted. The TCK model only reflects tensile damage. Regarding the strain rate effect, the KCC model shows a significant enhancement effect at high strain rates. The dynamic effects of the HJC and RHT models are not obvious, and the TCK model only considers the strain rate effect in the tensile case. The characteristics of the four models are summarized in Table 1.

## 3. Experiment and Numerical Scheme

To verify the suitability of four models under high-speed penetration, this section simulates the failure of reinforced concrete targets under high-speed impact by projectiles, and the simulation results are compared with the experimental results.

### 3.1. Experiment Set and Simulation Model

**Figure 6:**
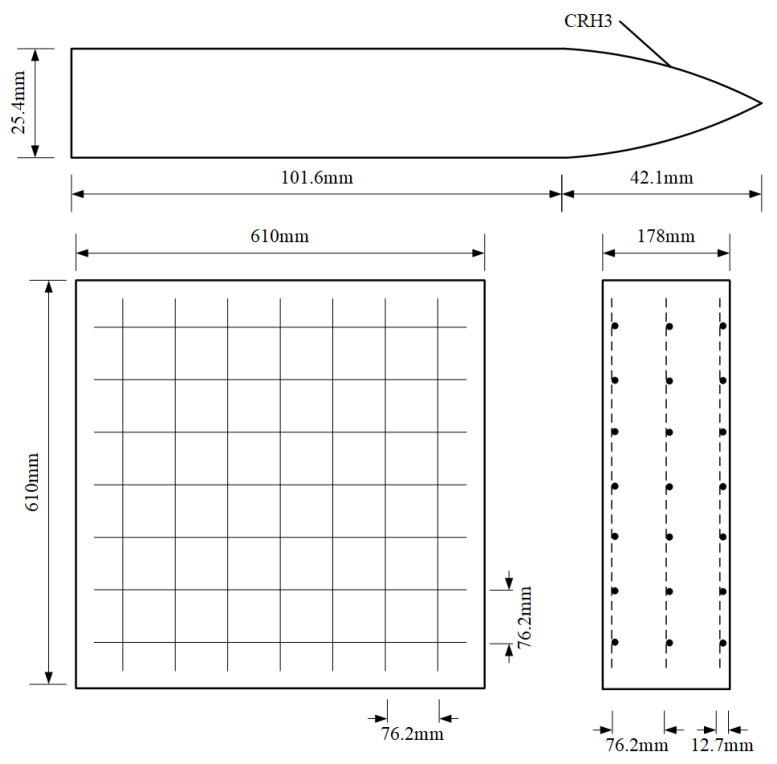
The target size and projectile used in the test.

The perforation experiments of reinforced concrete (RC) performed by Hanchak et al. [2] were adopted for the present analysis, as shown in Figure 6. The length of the projectile is 101.6 mm, the diameter is 25.4 mm, and the coefficient CRH of the oval head is 3. The dimension of the reinforced concrete target plate is 610 mm × 610 mm × 178 mm, the reinforcing steel bar is staggered in three layers, the mesh size is 76.2 mm × 76.2 mm, the distance between the steel layer and the concrete surface is 12.7 mm, and the diameter of the steel bar is 5.69 mm.

**Figure 7:**
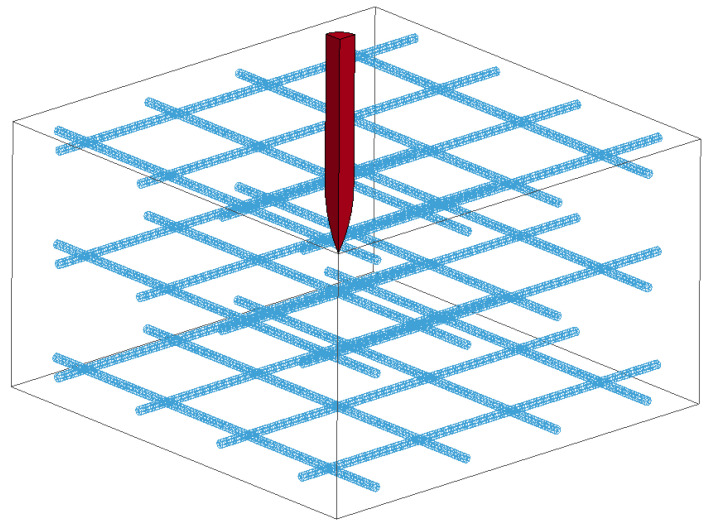
The numerical simulation model.

As shown in Figure 7, a 1/4 symmetry model was established, and normal constraints were applied to the symmetry plane. The projectile and concrete were a Lagrange grid, and the reinforcing steel bar was a beam grid. The interaction of the reinforcing steel bar and concrete was controlled by *CONSTRAINED_LAGRANGE_IN_SOLID. The contact type between the projectile and concrete was *ERODING_SURFACE_TO_SURFACE to reflect concrete failure.

The compressive strength of concrete in the test is 48 MPa, and the tensile strength is about 4 MPa. The HJC model is calibrated based on the literature [39,40,41], and the parameters of the RHT model were calibrated using [42]. The parameters of the KCC model were generated automatically based on strength. The TCK model was defined by the keyword *MAT_USER_DEFINED_MATERIAL_MODELS, and parameters were taken from [43]. The specific parameter sets are shown in Table 2, Table 3, Table 4 and Table 5.

The steel (T-250) projectile is modeled as a rigid part; the deformation of the projectile body can be ignored during the penetration process. The reinforcing steel bar adopted the MAT_PLASTIC_KINEMATIC model. The parameters are shown in Table 6 and Table 7.

In order to meet the requirement of calculation accuracy, a convergence analysis of the concrete grid was performed. When the grid size is less than 2.5 mm, the result is basically stable, so a 2 mm mesh size was used for calculation and analysis.

### 3.2. Basic Stress–Strain Analysis

The performance of each concrete model was first assessed using single-element tests of uniaxial loading.

**Figure 8:**
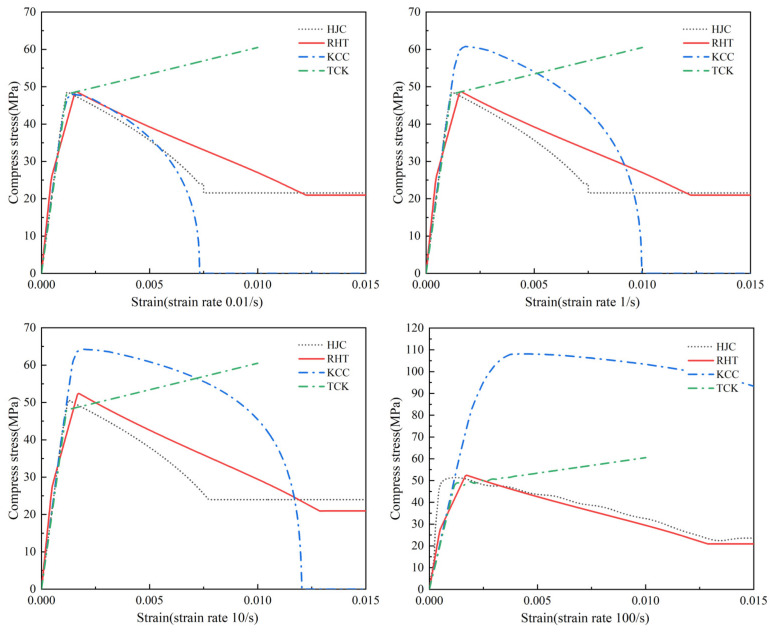
Uniaxial compression stress–strain curves at different strain rates (*f*_c_ = 48 MPa).

Figure 8 shows the stress–strain state of four models under uniaxial compression at different strain rates. In Figure 8, all four models simulate the ultimate strength of concrete accurately under quasi-static conditions. The HJC model and RHT model became perfectly plastic after reaching a softening point, the strength of the KCC model softened to a strength of 0, and the TCK model could not be used to describe the compressive damage and softening. When the strain rate is 1–100 s^−1^, the strain rate strengthening effect of KCC is significant, HJC and RHT models are not obvious, and the TCK model cannot reflect compressive strain rate strengthening.

**Figure 9:**
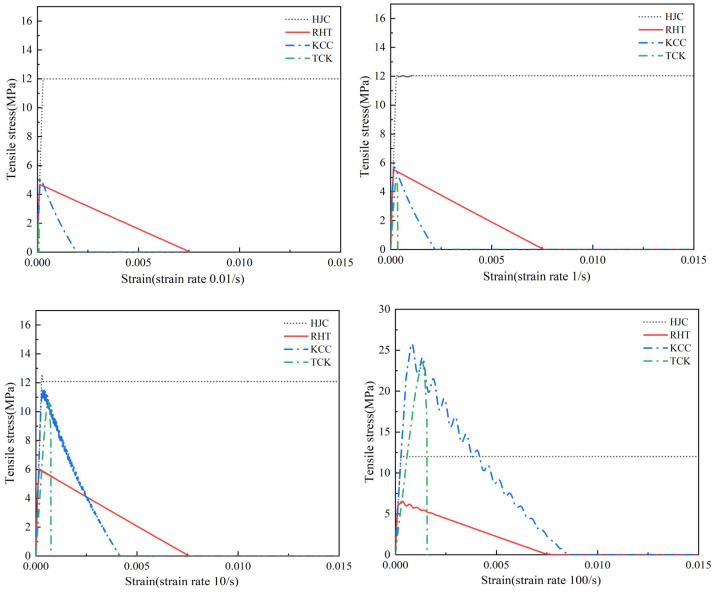
Uniaxial tensile stress–strain curves at different strain rates (*f*_t_ = 4 MPa).

Figure 9 shows the uniaxial tensile stress–strain relationship of the four models at different strain rates. The tensile section of the HJC model is elastic–perfectly plastic, and the softening stage is neglected, which may lead to serious underestimation of the tensile strain and the accumulation of damage. The RHT model is linear softening, the tensile fracture strain is fixed, and the strain rate strengthening effect is not obvious. The tensile softening of the KCC model is approximately linear, and the strength decay rate is almost constant. Due to the strain rate strengthening effect, the fracture strain increases significantly with the increase in the strain rate, which may greatly overestimate the fracture energy [18]. The softening stage of the TCK model is very short, and the strength rapidly decreases when the tensile limit is reached; since the damage of the model is only related to the degradation of the bulk modulus, the deviatoric stress state and stress–strain relationship of the material cannot be correctly described.

### 3.3. Material Failure and Erosion Algorithm

The erosion algorithm has been widely used in the numerical simulation of shock and explosion by deleting elements to characterize the failure of material [44]. Generally, the main purpose of the erosion algorithm is to avoid calculating termination caused by element distortion. At the same time, the erosion algorithm is also a common and effective technique used to characterize the perforation tunnel, cratering, and scrabbing [45,46,47,48].

Moreover, the erosion method is a method of characterizing severe damage. The process of deleting elements is irreversible. Therefore, the erosion criterion should be carefully introduced to capture the crushing (compressive failure) and fracture (tensile failure) of concrete.

It is generally accepted that compressive failure can be represented by the maximum principal strain [49]. Regarding tensile failure, the erosion criteria are still inconclusive in different models. The HJC model typically uses the minimum hydrostatic pressure for tensile failure [50]. Meanwhile, the maximum volumetric strain is always used in the RHT and KCC models [51]. In this study, the compression failure and tensile failure are controlled by maximum principal strain and maximum volumetric strain, respectively, with values of 0.15 and 0.003. The element erosion of the TCK model is directly determined by the critical damage value related to compressive plastic strain and tensile damage. The value of the compression and tensile damage criterion was determined empirically as 0.8 and 0.5 [43]. The values of compression erosion parameter *e_c_* and tensile erosion parameter *e_t_* are shown in Table 8. The effect of erosion parameters will be discussed in Section 4.2.

In the damage analysis in Section 4.1.1, only compression erosion is used to simulate the compression failure near the perforation tunnel, and other types of failure are represented by the damage value. In the crack analysis in Section 4.1.2, two types of erosion are used to simulate the failure of concrete.

## 4. Results and Discussion

### 4.1. Analysis of Simulation Results

#### 4.1.1. Damage Pattern Analysis

**Figure 10:**
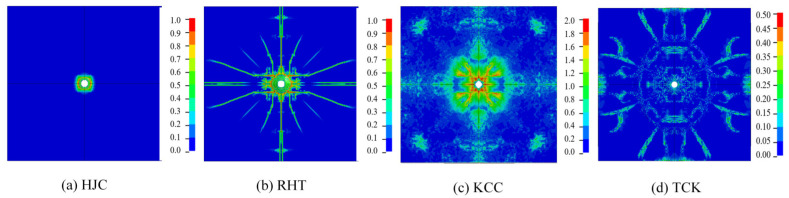
The damage pattern on the front face of the concrete targets.

**Figure 11:**
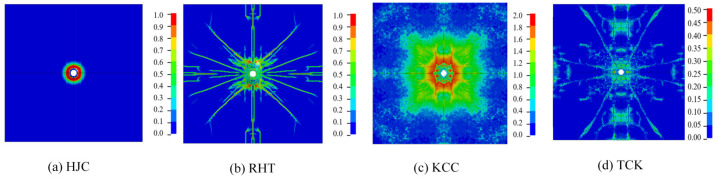
The damage pattern on the back face of the concrete targets.

**Figure 12:**
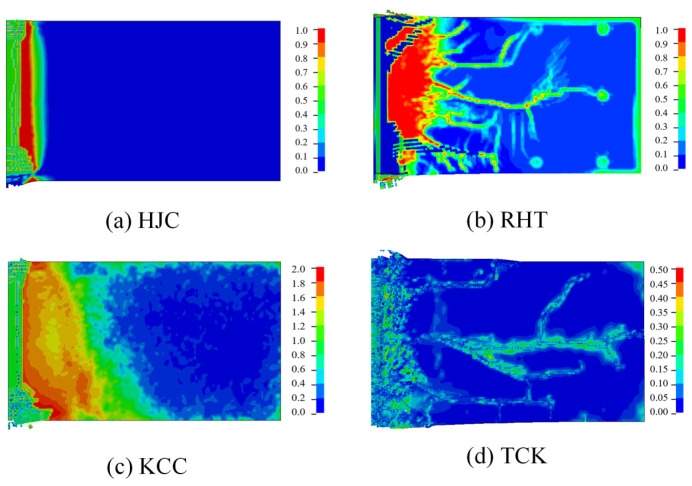
A section view of the damage pattern in concrete targets.

Figure 10, Figure 11 and Figure 12 illustrate the damage to the concrete target. The erosion algorithm eliminates severely distorted elements near the concrete perforation, and other failure patterns are characterized by the damage value.

Figure 10a shows that the damage of the HJC model is confined to the compressive-shear failure area near the perforation, with minimal tensile damage. This can be attributed to the mechanical properties discussed in Section 2.2.1. The tensile strength model of the HJC model assumes elastic–perfectly plastic behavior without considering softening caused by damage. Additionally, the strength surface does not account for the tension–compression ratio, leading to an overestimation of tensile strength and, consequently, slow accumulation of tensile plastic strain with almost no detection of tensile damage.

The simulation results of the RHT model, considering the influence of *J*_3_, effectively illustrate the compressive-shear damage and radial tensile damage near the perforation. For instance, the results show evidence of crushing near the perforation tunnel due to high pressure, radial cracks caused by the radial stress wave depicted in Figure 10b and Figure 11b, lamination cracks, breaks between concrete and the reinforcing steel bar, and the convergence of cracks within concrete in the symmetric plane, as illustrated in Figure 12b. However, the circumferential cracks representing cratering and scrabbing in the front and back faces cannot be observed. Because the softening curve of the tensile test is exponential decay, while the RHT model uses linear softening with slow decay at relatively low strain rates, the RHT model may overestimate the tensile fracture energy. The accumulation of damage near the concrete surface is slow, resulting in the absence of circular cracks on the surface when tensile damage extends to it.

The KCC model effectively captures cratering and scratching damage but does not clearly show surface cracks on the target. It separately calculates compressive and tensile damage but ultimately combines them into one value, making it difficult to directly assess the damage. Instead, the damage can only be indirectly expressed through the effective plastic strain. As a result, while the damage cloud map reflects the damage, it does not clearly indicate the extent of the damage, and it is ineffective in characterizing tensile failure.

The TCK model clearly reflects cratering, scrabbing, radial cracks, circular cracks, lamination cracks, and other types of damage to the concrete. However, the TCK model does not count the compression damage, so it cannot represent the compressive damage near the perforation through the damage value. The damage near the tunnel in Figure 12d is caused by the tensile wave generated by unloading after perforation rather than compression in penetration progress.

#### 4.1.2. Failure Pattern Analysis

**Figure 13:**
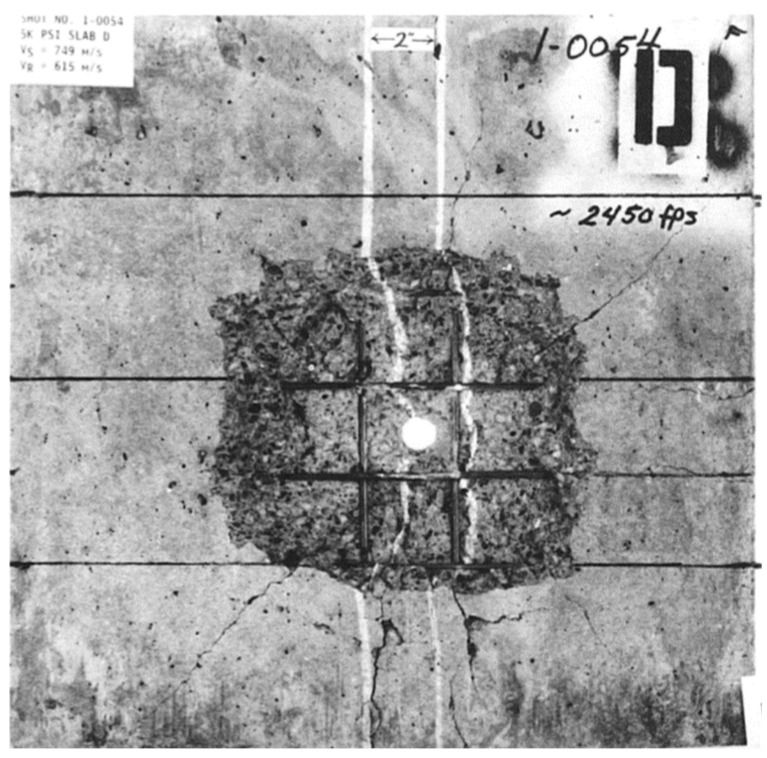
The experiment results of the front face [34].

In this section, the erosion algorithm is used to simulate all macroscopic failures of concrete. The experimental results of the front face are depicted in Figure 13, showing an almost square cratering area. Numerous radial cracks extend from the cratering area to the edge of the target plate, some of which are bifurcated. Apart from oblique cracks at a 45° angle, the remaining cracks are located near the reinforcing steel bar. The depth of the cratering area is approximately one-third of the target thickness.

**Figure 14:**
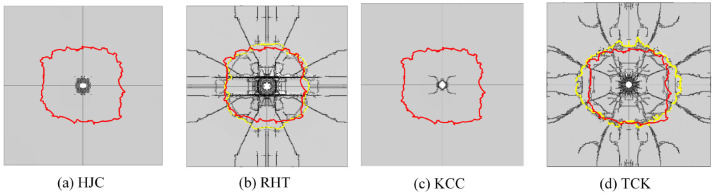
The simulation results of the failure pattern on the front face.

**Figure 15:**
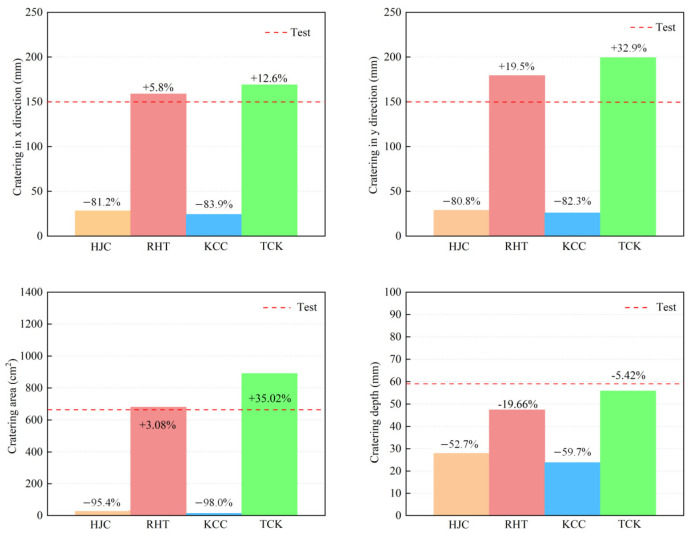
A comparison of the simulation and test results of the front cratering range.

Figure 14 shows the damage patterns on the front face. The solid red line represents the cratering profile in the test, while the dashed yellow line depicts the cratering area in the simulation. Figure 15 illustrates the comparison between the simulation and test values for the cratering range in the x direction, y direction, cratering area, and cratering depth. Among these, the HJC and KCC models do not exhibit any cracks, and the cratering area is confined to the vicinity of the perforation tunnel. The RHT model accurately portrays the cratering range, radial cracks, and spalling failure near the reinforcing steel bar, with an error in the estimation of the cratering area of just 3.08%. The TCK model reflects the cratering area and radial cracks, with noticeable crack bifurcation and an overestimation of the cratering range. Consequently, the RHT model yields the most accurate simulation result for frontal damage, followed by the TCK model. The simulation error of the KCC model and HJC model is too large for practical use.

**Figure 16:**
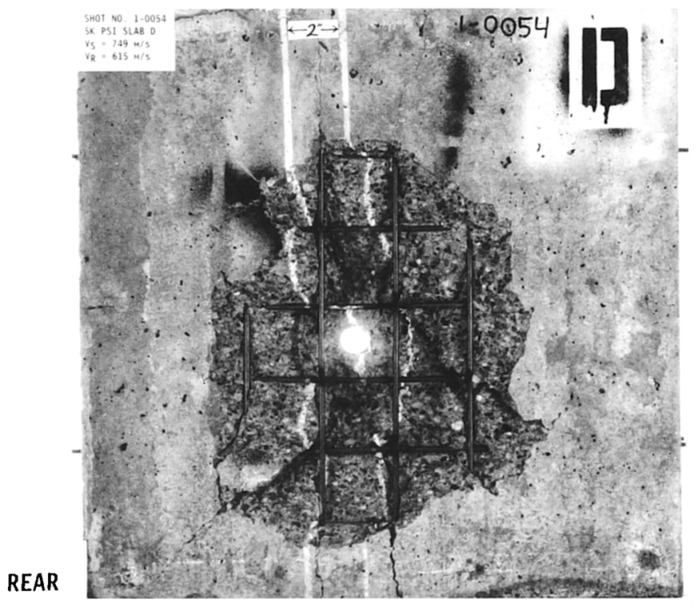
The experimental results of the back face [34].

**Figure 17:**
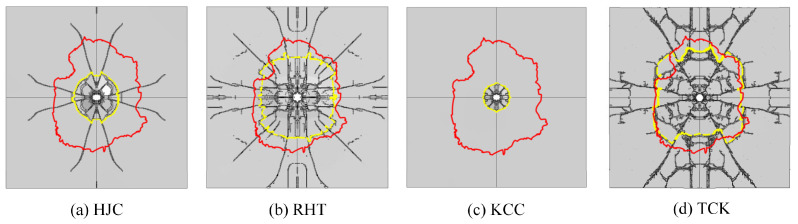
The simulation results of the failure pattern on the back face.

**Figure 18:**
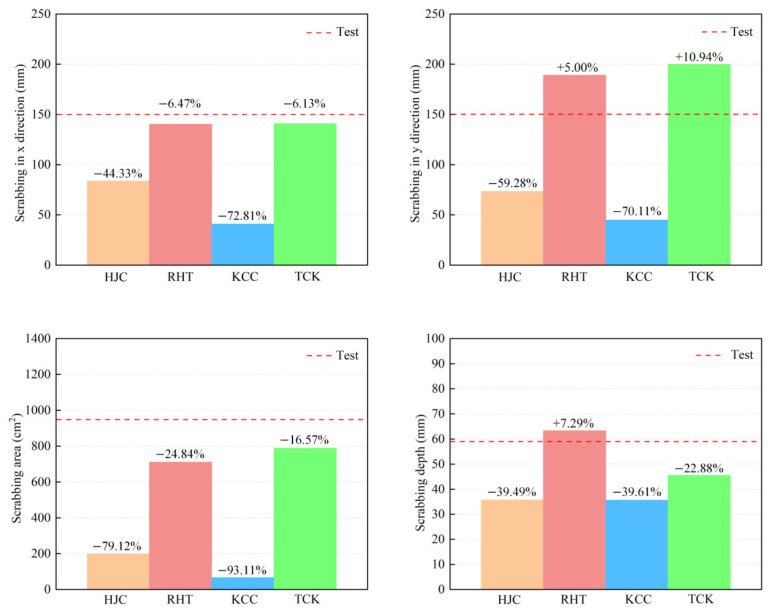
A comparison of the simulation and test results of the back scrabbing range.

The scrabbing area, shown in Figure 16, features an irregular shape with a small number of cracks along the central reinforcing steel bar. The depth of the scrabbing area is about one-third of the target thickness. Figure 17 and Figure 18 display the simulation results and a comparison with test values.

The HJC model shows an approximately circular scrabbing area, with radial cracks extending to the edge of the target. The RHT model’s scrabbing range is slightly smaller than the test value, with radial and oblique cracks near the reinforcing steel bar. The KCC model has a very small back scrabbing area without cracks. The TCK model accurately simulates the scrabbing area shape and all types of cracks. In comparing the models, the RHT model has the smallest error, followed by the TCK model, while the estimations of the HJC and KCC models are inaccurate.

**Figure 19:**
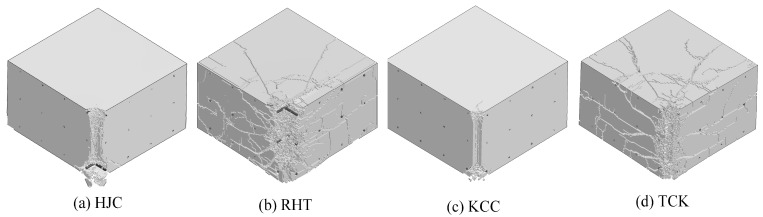
An isometric view of the overall failure pattern.

The overall failure pattern of the concrete target is shown in Figure 19. The failure area of the HJC and KCC models is concentrated near the perforation, and the scrabbing area is slightly larger than the cratering area. The RHT model and TCK model can not only clearly reflect the cratering and scrabbing but also reflect the internal lamination crack.

The macroscopic phenomena observed in the simulation can be explained through the analysis presented in Section 3. The tensile strength model of the HJC is elastic–perfectly plastic, which disregards the softening stage. The tensile strain is underestimated, which limits the expansion of tensile cracks. Consequently, the range of cratering and scrabbing and the growth of radial cracks are almost invisible.

On the other hand, the RHT and KCC models consider the tensile softening of the material, with the softening function being approximately linear. The maximum tensile strain failure criterion, in combination with this softening, should be able to clearly reflect all kinds of tensile failure of concrete. However, the KCC model’s strengthening factor of tensile stress at a high strain rate is much higher than that of the RHT model, potentially leading to an overestimation of the absorption of fracture energy. Therefore, the element deformation is small, which makes it difficult for the KCC model to simulate crack propagation with the erosion algorithm.

Furthermore, the TCK model uses the density of cracks per unit volume to reflect the tensile damage of the material. The development of element failure follows the nucleation–convergence–propagation of microcracks, with the initial density of cracks within the element following a Weibull random distribution. This approach leads to a failure pattern that is more consistent with the real situation.

In summary, when using the erosion algorithm to describe concrete cracks being penetrated, the RHT and TCK models provide better simulation results. Regarding penetration, the KCC model may require the selection of an appropriate strain rate enhancement factor, while the HJC model is unsuitable for describing concrete failure induced by perforation.

#### 4.1.3. Residual Velocity Analysis

**Figure 20:**
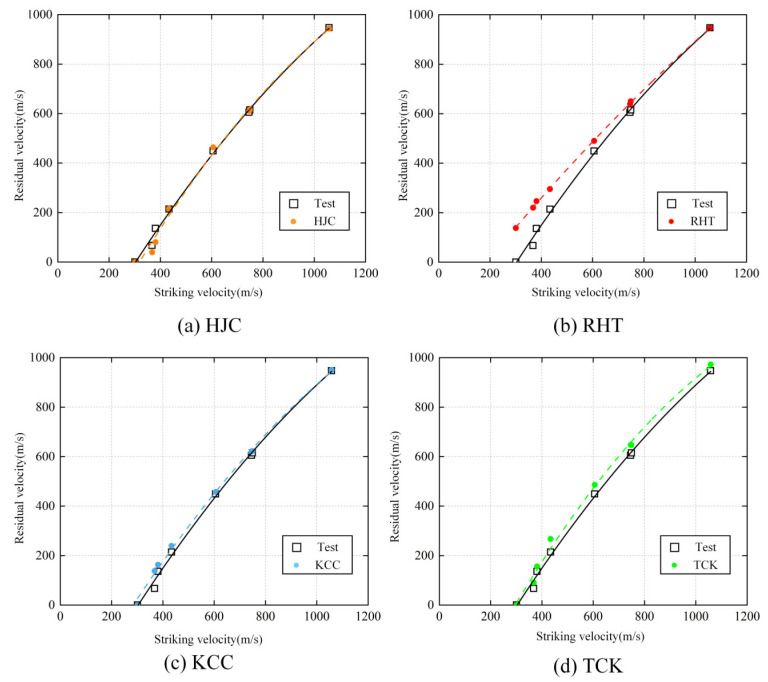
The residual velocity at different conditions.

The prediction of the residual velocity of projectiles in different conditions is shown in Figure 20, and the estimation of four concrete constitutive models is compared with the test result.

In the velocity range of 300 m/s to 1100 m/s, the prediction results of the four models closely align with the test results. Particularly, the HJC and KCC models accurately depict the residual velocities of projectiles penetrating reinforced concrete targets at various initial velocities. However, when using the RHT model to simulate low-velocity penetration, the simulated residual velocity tends to be higher than the actual test values. As the velocity increases, the simulated values gradually converge toward the test results. Notably, all prediction results from the TCK model consistently exceed the test values.

The discrepancy in the RHT model can be attributed to the compression erosion parameters calibrated for high-velocity penetration, which may not be suitable for low-velocity penetration. This leads to reduced target resistance and the rapid deletion of elements with large residual stress. On the other hand, the TCK model’s error stems from the neglect of strain strengthening and the material’s compressive strain rate effect. As a projectile penetrates the concrete, the stress calculated by the ideal elastic–plastic model is lower, thereby reducing resistance to the projectile. Furthermore, as the projectile approaches the rear side of the concrete target, failure caused by tensile damage results in the premature failure and deletion of elements before the projectile even reaches them, further diminishing the concrete’s resistance force.

### 4.2. Parameter Study of Erosion

The erosion algorithm, as explained in Section 3.3, has a substantial impact on the outcome of the penetration simulation. Therefore, this section discusses the sensitivity of erosion parameters *e_c_* and *e_t_*. Considering the simulation results at 749 m/s and the erosion parameters in Section 3.3, we establish a variation range for one parameter at ±25% and ±50% while keeping the other parameter fixed. The analysis focuses on examining the influence of altering erosion parameters on the cratering area, cratering depth, scrubbing area, scrubbing depth, and residual velocity.

**Figure 21:**
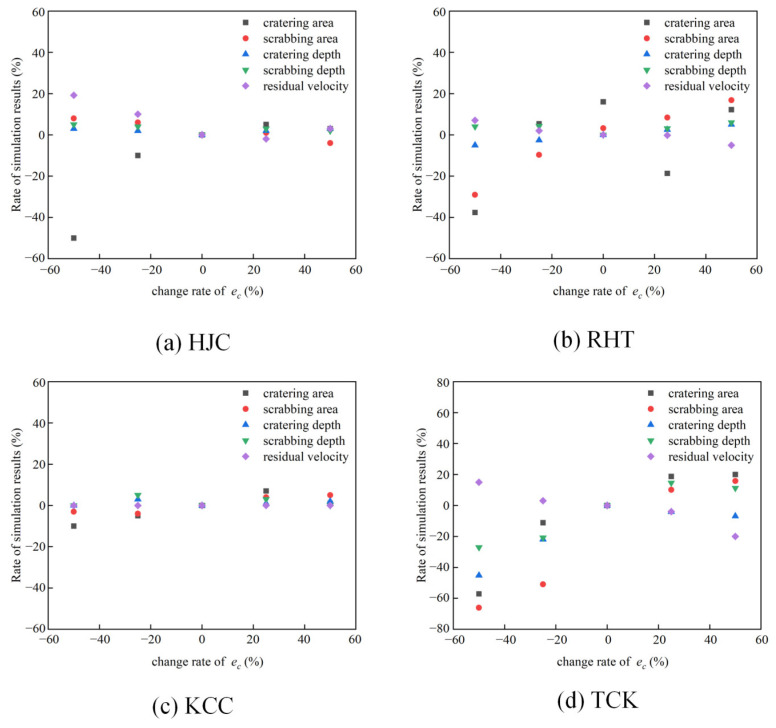
The effect of *e_c_* on the simulation results of the penetration test.

In Figure 21, the impact of the compression erosion parameter (*e_c_*) on the simulation results is depicted. Across the four models, the residual velocity of the projectile diminishes as the compressive erosion increases. When the deviation range of compressive erosion is below 25%, the residual velocity remains relatively stable. However, when the deviation exceeds 50%, significant changes in the results are evident. The cratering and scrabbing of the target exhibit sensitivity to the compression erosion parameters. For deviations less than −25%, the cratering and scrabbing range notably decreases. This is attributed to the small compression erosion parameters, which prompt the rapid deletion of elements near the projectile’s head, disrupting the stress path and normal propagation of stress waves, thereby leading to a lack of reflection in cratering and scrabbing.

**Figure 22:**
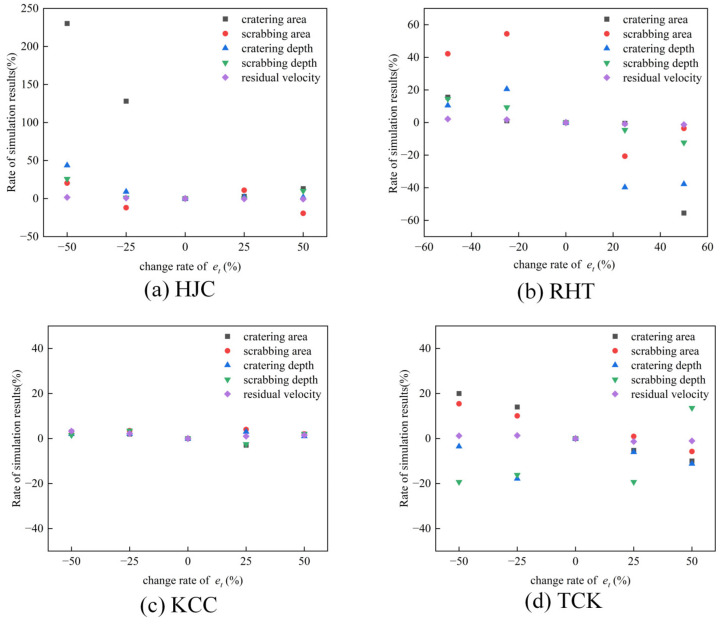
The effects of *e_t_* on the simulation results of the penetration test.

Figure 22 illustrates the effects of the tensile erosion parameter (*e_t_*) on the results of the penetration simulation. Both the HJC and RHT models exhibit high sensitivity to changes in *e_t_*, particularly in the cratering and scrubbing areas, leading to significant and irregular differences in the simulation results. However, the tensile erosion criterion does not noticeably affect the simulation results of the KCC model. Regarding the TCK model, the cratering and scrabbing area decreases as the tensile erosion threshold increases. Remarkably, the tensile erosion criterion has almost no influence on the prediction of the residual velocity of the projectile.

The erosion threshold for the HJC and RHT models is based on strain and is not related to damage evolution. As a result, the failure of elements is abrupt, leading to unstable simulation results. On the other hand, for the TCK model, the deletion of elements is directly controlled by the damage evolution equation, ensuring the accuracy of the residual velocity of the projectile and the failure of concrete. The simulation results for the TCK model exhibit strong regularity.

It should be noted that the stress–strain state in the concrete target is highly complex during cratering and scraping stages. The failure is influenced by both compression and tension, making it challenging to accurately describe using a single erosion criterion. Using an excessively small erosion criterion will result in the deletion of many elements with significant residual stress, thereby underestimating the structure’s resistance to the projectile and disrupting the propagation of stress waves. Therefore, while the erosion criterion can be used to describe the macroscopic cracks of concrete, it should be carefully selected and validated.

## 5. Conclusions

The accuracy of the numerical prediction of responses of concrete structures subjected to high-speed impact depends on the material models used in the analysis. This study reviewed four commonly used concrete material models. The accuracy and suitability of the models in simulating concrete structure responses were discussed through comparisons of the simulation results with test data. The following conclusions can be drawn:

(1) The RHT and KCC models are theoretically complete in both tension and compression. In contrast, the HJC model is inaccurate in describing the tensile state of concrete, and the TCK model is relatively simplified in describing the compression characteristics.
(2) The RHT and TCK models can effectively describe all types of failure in concrete through damage value or erosion. The HJC model inaccurately represents tensile damage and crack propagation, while the KCC model’s damage pattern for estimating cracks is blurred and can barely simulate tensile cracks through erosion.
(3) The record of residual velocity shows that HJC and KCC have more accurate estimations at different initial speeds; the RHT model tends to overestimate the residual velocity of low-velocity penetration, while the TCK model overestimates residual velocity in all velocity conditions.
(4) The effects of erosion parameters are discussed using sensitivity analysis. The residual velocity of the projectile is mainly affected by compressive erosion, while the cratering and scrabbing stages are affected by both tensile and compressive erosion. Strain-based erosion tends to lead to unstable simulation results in the HJC, RHT, and KCC models, whereas damage-based erosion demonstrates better stability and regularity in the TCK model.
(5) Our study has potential limitations. This study compares the theories of several common concrete material models and their different properties indicated in a high-speed perforation simulation. However, due to the different structures and materials of the target slabs and impact conditions, suitability research using models is still challenging and varies widely. Therefore, the results of different tests, including impacts and blasts, will be presented in future works.

## Figures and Tables

**Table 1:** A summary of material models for concrete and its properties.

Model	Strength Surface		DIF		EOS	Failure Criterion	Damage	
	Quantity	*J*_3_ Effect	Compress	Tensile			Compress	Tensile
HJC	1	No	Yes	No	Yes	Yes	Yes	No
RHT	3	Yes	Yes	Yes	Yes	Yes	Yes	Yes
KCC	3	Yes	User-defined	User-defined	Yes	No	Yes	Yes
TCK	1	No	No	Yes	No	Yes	No	Yes

**Table 2:** The parameters of the HJC model.

Parameter	*ρ* (g·cm^−3^)	*G* (GPa)	*A* (MPa)	*B* (MPa)	*C*	*N*	*f*_c_ (MPa)	*T* (MPa)	*S*_max_	EFMIN
value	2.44	1.49	0.27	1.86	0.007	0.84	48	4.2	7.0	0.01
parameter	*P*_crush_ (MPa)	*μ*_crush_	*P*_lock_ (MPa)	*μ*_lock_	*D*_1_	*D*_2_	*K*_1_ (GPa)	*K*_2_ (GPa)	*K*_3_ (GPa)	
value	0.16	0.001	80	0.1	0.04	1	62	−40	26	

**Table 3:** The parameters of the RHT model.

Parameter	*ρ* (g·cm^−3^)	*G* (GPa)	*B*_0_	*B*_1_	*T*_1_ (MPa)	*A_f_*	*N_f_*	*f*_c_ (MPa)	*f*_s_*	*f*_t_*
value	2.44	1.49	1.22	1.22	3.527 × 10^4^	1.60	0.61	48	0.18	0.066
parameter	*A*_1_ (MPa)	*A*_2_ (MPa)	*A*_3_ (MPa)	*P*_el_ (MPa)	*P*_co_ (MPa)	*D*_1_	*D*_2_	EPM	*T*_1_ (MPa)	*T*_2_ (MPa)
value	3.527 × 10^4^	3.958 × 10^4^	9.04 × 10^3^	23.3	6 × 10^3^	0.04	1.0	0.01	3.527 × 10^4^	0
parameter	*E*_0C_	*E*_0T_	*E_c_*	*E_t_*	*β_c_*	*β_t_*	PTF	g_c_*	g_t_*	*ξ*
value	3 × 10^−11^	3 × 10^−12^	3 × 10^19^	3 × 10^19^	0.025	0.001	0.001	0.53	0.07	0.5

**Table 4:** The parameters of the KCC model.

Parameter	*ρ* (g·cm^−3^)	*μ*	*A*_0_ (MPa)	*f*_t_ (MPa)	*R*_size_ (Inches/m)	UCF (psi/MPa)
value	2.44	0.2	−48	4	39.37	145

**Table 5:** The parameters of the TCK model.

Parameter	*ρ* (g·cm^−3^)	*σ*_y_ (MPa)	*Kic* (MPa·m^1/2^)	*E* (GPa)	*μ*	*m*	*k*
value	2.44	48	2.74	20.68	0.2	6	0.1077

**Table 6:** The parameters of the MAT_RIGID model.

Parameter	*ρ* (g·cm^−3^)	*E* (GPa)	*μ*	*σ* (GPa)	*ε*
value	8.02	210	0.3	1.72	0

**Table 7:** The parameters of the PLASTIC_KINEMATIC model.

Parameter	*ρ* (g·cm^−3^)	*E* (GPa)	*μ*	SIGY (GPa)	RTAN (GPa)
value	7.86	210	0.3	0.4	0.76

**Table 8:** The erosion criterion.

Model	HJC		RHT		KCC		TCK	
Erosion	*e_c_*	*e_t_*	*e_c_*	*e_t_*	*e_c_*	*e_t_*	*e_c_*	*e_t_*
Value	0.15	0.003	0.15	0.003	0.15	0.003	0.8	0.5

## Data Availability

The original contributions presented in the study are included in the article, further inquiries can be directed to the corresponding author.
